# Early laboratory indicators of acute metabolic decompensation during emergency presentations in pediatric maple syrup urine disease

**DOI:** 10.1007/s00431-026-07081-4

**Published:** 2026-05-19

**Authors:** Sinem Oral-Cebeci, Çiğdem Aktuğlu-Zeybek, Beyza Aslan, Özge Yaren Uysal, Mehmet Şerif Cansever, Ertuğrul Kıykım, Tanyel Zubarioglu

**Affiliations:** 1 Department of Pediatric Emergency, Cerrahpaşa Medical Faculty, İstanbul University-Cerrahpaşa, Istanbul, Turkey; 2 Department of Pediatric Nutrition and Metabolism, Cerrahpaşa Medical Faculty, İstanbul University-Cerrahpaşa, Istanbul, Turkey; 3 Research Laboratory of Metabolism, Cerrahpaşa Medical Faculty, İstanbul University-Cerrahpaşa, Istanbul, Turkey; 4 Department of Pediatrics, Cerrahpaşa Medical Faculty, İstanbul University-Cerrahpaşa, Istanbul, Turkey; 5 Division of Medical Laboratory Techniques, Department of Medical Documentation and Techniques, The Vocational School of Health Services, İstanbul University-Cerrahpaşa, Istanbul, Turkey

**Keywords:** Maple syrup urine disease, Pediatric emergency medicine, Acute metabolic decompensation, Hyperuricemia, Biomarker

## Abstract

Acute metabolic decompensation (AMD) is the most life-threatening complication of maple syrup urine disease (MSUD). Recognizing AMD early in the emergency department (ED) is difficult because the most important clinical indicators—neurological manifestations—typically appear late during decompensation, and plasma leucine measurements are frequently delayed. This study aimed to identify laboratory biomarkers that can be obtained quickly and support early AMD detection at ED. This retrospective study included MSUD patients aged 28 days–18 years admitted to a tertiary pediatric ED between 2016 and 2025. Acute neurological deterioration, reduced oral intake, or vomiting with ketosis or plasma leucine levels ≥ 600 µmol/L were defined as AMD. Presenting complaints, duration of hospitalization, treatment modalities, and laboratory investigations were recorded and compared between ED visits with AMD and non-AMD. Among 25 patients, 269 ED visits were analyzed; 79 (29.4%) met AMD criteria. AMD visits were associated with worse clinical outcomes, including higher rates of pediatric intensive care admissions (22% vs. 2.6%) and increased hemodialysis requirements (19% vs. 1.6%). Hyponatremia (*p* = 0.005) and hyperuricemia (*p* < 0.001) were more prevalent during AMD. Acid–base parameters did not significantly discriminate AMD. Among primary laboratory tests, only serum uric acid was a clinically meaningful predictor (AUC 0.786). A cutoff value of 4.65 mg/dL for uric acid was identified to predict AMD.

*Conclusion*: Serum uric acid is a reliable and clinically practical early predictor of AMD. This readily available marker may enable earlier risk stratification and intervention when leucine results are delayed, potentially improving outcomes in pediatric MSUD.

**Table:** 

**What is Known:** • *Acute metabolic decompensation (AMD) in pediatric maple syrup urine disease (MSUD) can rapidly lead to encephalopathy, cerebral edema, and permanent neurologic injury.* **•** *Early emergency presentations are clinically nonspecific, and plasma leucine—the diagnostic gold standard—is often not available in real time.*	
**What is New:** • *A cutoff value of 4.65 mg/dL for uric acid was identified to predict AMD.* • *Respiratory symptoms and fever were significantly more frequent in non-AMD visits, suggesting that infectious symptoms alone do not necessarily indicate metabolic instability in MSUD patients.*

**Supplementary Information:**

The online version contains supplementary material available at 10.1007/s00431-026-07081-4.

## Introduction

Maple syrup urine disease (MSUD) is an autosomal recessive inherited metabolic disorder caused by deficiency of the branched-chain α-keto acid dehydrogenase (BCKAD) complex, which is required for the catabolism of branched-chain amino acids (BCAAs): leucine, isoleucine, and valine [1]. The BCKAD complex consists of the catalytic subunits E1α (BCKDHA), E1β (BCKDHB), E2 (DBT), and E3 (DLD) [2]. MSUD is clinically classified into classic, intermediate, intermittent, thiamine-responsive, and rare E3-deficient forms [2]. The classic subtype is the most severe and presents in the neonatal period, while other forms show variable residual enzyme activity and clinical severity [3–5].

Diagnosis is established by detecting elevated plasma BCAAs—particularly leucine—and corresponding branched-chain α-ketoacids, along with the presence of alloisoleucine [4]. Diagnostic confirmation is made by identification of biallelic pathogenic variants in BCKDHA, BCKDHB, DBT, or DLD [6]. Prognosis largely depends on early diagnosis and strict metabolic control, especially in the classic form, where timely intervention significantly improves neurodevelopmental outcomes [7].

All individuals with MSUD remain at risk for acute metabolic decompensation (AMD) during catabolic stress such as infection, fever, fasting or surgery [8]. AMD management aims to suppress catabolism and promote anabolism through increased caloric intake, temporary protein restriction, administration of BCAA-free amino acid mixtures supplemented with isoleucine and valine, and, when necessary, intravenous glucose, lipid support, and insulin infusion [3, 9–12]. Oral sodium phenylbutyrate may be used as adjunctive therapy in selected cases [13], while hemodialysis is reserved for severe or refractory episodes [14].

Although therapeutic strategies for AMD are well established, early recognition in the emergency department (ED) remains challenging. Initial symptoms are often nonspecific, and plasma leucine measurements are not universally or rapidly available, potentially delaying targeted treatment [15]. It has been suggested that routine biochemical parameters including metabolic and acid–base markers may assist in early identification of AMD [15]. However, evidence defining reliable early predictors is limited.

The aim of this study was to identify accessible and rapidly obtainable laboratory biomarkers that support early diagnosis of AMD in MSUD patients presenting to pediatric EDs with nonspecific symptoms. By focusing on primary laboratory parameters available during initial evaluation, we aim to facilitate timely treatment initiation and optimize emergency resource utilization.

## Materials and methods

### Study design and participants

This retrospective study was conducted in the Pediatric Emergency Department of a tertiary referral center for inherited metabolic diseases and included patients admitted between June 2016 and June 2025. Patients followed in the Department of Nutrition and Metabolism with a molecularly and/or biochemically confirmed diagnosis of MSUD were eligible.

Participants were aged 28 days to 18 years and presented to the pediatric ED with clinical findings consistent with AMD, as defined in the methodology. Only cases with complete clinical and laboratory data were included. Patients were excluded if they had irregular outpatient follow-up, poor treatment adherence, prior liver transplantation, or additional chronic conditions that could affect laboratory parameters, such as chronic kidney disease or hepatic insufficiency. Ethical approval was obtained from the local ethics committee (approval number E-24687260–604.01–1407328), and informed consent was obtained from the parents of all participants.

### Data collection

Patient characteristics, including demographic data, molecular and biochemical diagnostic findings, time of diagnosis, ongoing medical and nutritional therapies, and secondary comorbidities, were recorded. At ED admission, presenting complaints, neurological findings, hospitalization duration, treatments administered, metabolic attack course, and laboratory results were documented.

Symptom variables were defined at presentation using standardized clinical criteria. Fever was defined as a measured body temperature of ≥38.0 °C using an age-appropriate technique [16]. Altered consciousness was defined as a non-alert response on the alert, verbal, pain, unresponsive (AVPU) scale, a pediatric Glasgow Coma Scale score <15, or a newly observed deviation from baseline mental status, including lethargy, difficulty with arousal, confusion, or decreased responsiveness [17]. Respiratory tract involvement was categorized as upper or lower respiratory findings. Upper respiratory symptoms included rhinorrhea, nasal congestion, sore throat, or acute cough. Lower respiratory findings included cough with age-adjusted tachypnea, nasal flaring, grunting, chest wall retractions, wheezing, crackles, or oxygen saturation ≤95% on room air.

The laboratory parameters studied included quantitative plasma amino acid analysis, including BCAA levels and routine biochemical markers: ammonia, pH, pCO_2_, HCO_3_^−^, lactate, uric acid, urea, creatinine, glucose, alanine aminotransferase (ALT), aspartate aminotransferase (AST), alkaline phosphatase (ALP), gamma-glutamyl transferase (GGT), electrolytes, anion gap, and urine ketones.

An elevated anion gap was defined as [Na^+^ − (Cl^−^ + HCO_3_^−^)] > 16 mEq/L. Urine ketones (assessed by dipstick test) and α-keto acids (measured by dinitrophenylhydrazine (DNPH) test) were semi-quantitatively categorized as negative, 1+, 2+, 3+, or 4+ based on standard visual scales. All laboratory samples analyzed in this study were obtained at the time of initial presentation to the pediatric ED, before the initiation of emergency treatment, including parenteral nutritional support, insulin, or extracorporeal therapies.

### The definition of acute metabolic decompensation

Acute metabolic decompensation in patients with MSUD was defined as the presence of at least one of the following clinical criteria, without an alternative medical explanation:

1. Acute onset of altered mental status (confusion, lethargy or coma) or the appearance of new-onset neurologic symptoms, including but not limited to ataxia, dystonia, choreoathetosis, or seizures
2. Acute reduction in oral intake or vomiting, accompanied by either:
  Ketonuria or ketoaciduria, defined as ≥ 1 + on urine dipstick testing in infants or ≥ 2 + in older children
  An elevated plasma leucine level of more than 600 µmol/L

### Emergency education and sick-day management

All patients in this cohort were followed at a single tertiary referral center for inherited metabolic diseases and managed according to a standardized institutional protocol. As part of routine care, patients and caregivers received structured education on early recognition of catabolic stress and emergency management. Families were instructed to present promptly for medical evaluation if fever, vomiting, decreased oral intake, prolonged fasting, lethargy, reduced alertness, imbalance, or any new neurological symptom potentially suggestive of metabolic instability occurred. Caregivers were also trained to perform urine ketone testing at home.

Patients received a written emergency letter recommending early referral to the pediatric ED even with mild symptoms, to prevent progression of catabolism. This emergency document also included guidance for emergency physicians regarding the initial laboratory evaluation and metabolic management approach for suspected AMD.

### Statistical analysis

Data analysis was conducted in the R environment (v. 4.4.2). Normality of continuous variables was assessed using the Kolmogorov–Smirnov and Shapiro–Wilk tests, supported by visual inspection of Q–Q plots and histograms. Summary measures for continuous data are reported as median (range), while categorical variables are presented as frequency (%). Group differences were assessed using the Mann–Whitney *U* test for continuous variables and Pearson’s chi-squared test or Fisher’s exact test for categorical variables. Receiver operating characteristic (ROC) curve analysis was performed to evaluate the discriminative ability of each laboratory parameter for predicting AMD status. Area under the curve (AUC) values with 95% confidence intervals were calculated using DeLong’s method; an AUC significantly different from 0.5 indicated discriminative ability (*p* < 0.05). Calibration was assessed using Brier scores (lower values indicate better calibration) and Hosmer–Lemeshow goodness-of-fit tests (*p* > 0.05 indicates adequate calibration). Optimal cutoff points were determined using Youden’s index. Firth’s penalized logistic regression was used due to the small number of cases and to address the issue of separation. Modeling began with univariate screening of all potential predictors, selecting those with a statistically significant *p*-value for inclusion in the multivariable model, and including age and sex to control for potential confounding, regardless of their univariate significance. To address multicollinearity, the variance inflation factor (VIF) was calculated for all included predictors, and variables with a VIF greater than 10 were iteratively removed until all remaining variables met the threshold. The final model was subjected to diagnostic checks, including testing the linearity assumption by visual examination and assessing for influential data points and outliers using Cook’s distance and standardized deviance residuals. A two-sided *p*-value < 0.05 was considered statistically significant.

## Results

### Study cohort

The study cohort included 25 patients with MSUD, accounting for 269 pediatric ED visits during the study period. The population was predominantly male (64%), with a median age of 1.8 years (range 0.0–17.8), and 68% of visits occurred in children younger than five years. All patients were receiving protein-restricted medical nutrition therapy consisting of BCAA-free amino acid mixtures supplemented with valine and isoleucine to maintain plasma BCAA concentrations within therapeutic targets. Feeding was primarily oral (88%), while 11% required percutaneous endoscopic gastrostomy and 0.7% nasogastric tube feeding. Age distribution and feeding modalities are summarized in Supplementary Material S1, and detailed demographic and genotypic characteristics are provided in Supplementary Material S2.

Patients had a median of 7 ED admissions (range 1–28). Of the 269 visits, 79 (29.4%) were associated with AMD, while 190 (70.6%) occurred without AMD. Median age at presentation did not differ significantly between AMD and non-AMD visits (4.9 vs. 4.1 years, *p* = 0.122).

### Distribution of clinical findings, treatment features, and outcomes according to AMD status

Symptoms were present in 82% of pediatric ED visits; the remaining asymptomatic cases were referrals prompted by routine plasma amino acid analysis showing leucine levels above 600 µmol/L. Because acute neurological findings were included in the predefined diagnostic criteria for AMD, visits presenting with neurological symptoms were classified as AMD episodes and excluded from comparative analyses.

**Fig. 1:**
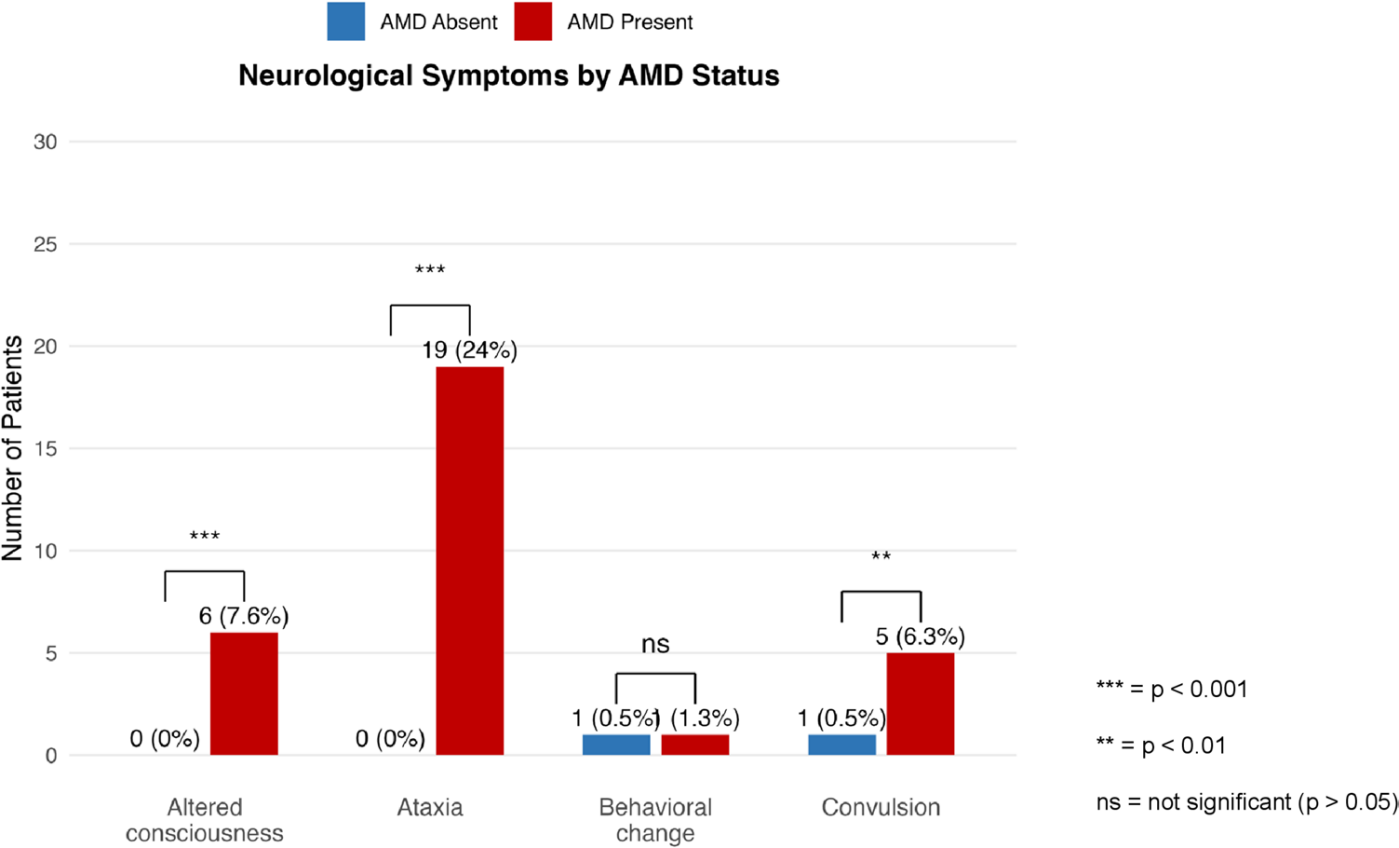
Distribution of neurological manifestations observed during AMD-associated emergency visits

Among AMD-associated visits, neurological manifestations were observed in 54%, including ataxia (24%), altered consciousness (7.6%), and convulsions (6.3%). In contrast, several non-neurological features differed between groups. Respiratory tract symptoms (32% vs. 3.8%, *p* < 0.001) and fever (14% vs. 5.1%, *p* = 0.032) were more frequent in non-AMD visits. Other nonspecific symptoms—vomiting, decreased oral intake, malaise and diarrhea—did not differ significantly. The distribution of clinical symptoms by AMD status is shown in Table 1, and neurological manifestations during AMD episodes are illustrated in Figure 1.

**Table 1:** Clinical symptoms at pediatric emergency department admission by AMD status

	Presence among all PED visits *N* = 269^a^	AMD absent *N* = 190^a^	AMD present *N* = 79^a^	*p*-value
Presence of symptoms at admission	221 (82)	161 (85)	60 (76)	0.086^b^
Fever	31 (12)	27 (14)	4 (5.1)	**0.032**^**b**^
Vomiting	49 (18)	32 (17)	17 (22)	0.365^b^
Poor feeding	25 (9.3)	18 (9.5)	7 (8.9)	0.875^b^
Malaise	22 (8.2)	17 (8.9)	5 (6.3)	0.475^b^
Respiratory tract symptoms	64 (24)	61 (32)	3 (3.8)	** < 0.001**^**b**^
Diarrhea	10 (3.7)	8 (4.2)	2 (2.5)	0.728^c^
Abdominal pain	1 (0.4)	1 (0.5)	0 (0)	> 0.999^c^
Rash	1 (0.4)	1 (0.5)	0 (0)	> 0.999^c^
Other	20 (7.4)	16 (8.4)	4 (5.1)	0.339^b^

^a^Median (min–max); *n* (%), ^b^Pearson’s chi-squared test, ^c^Fisher’s exact test

Bold values indicate statistically significant *p*-values (*p *< 0.05)

*PED*, pediatric emergency department; *AMD*, acute metabolic decompensation

**Fig. 2:**
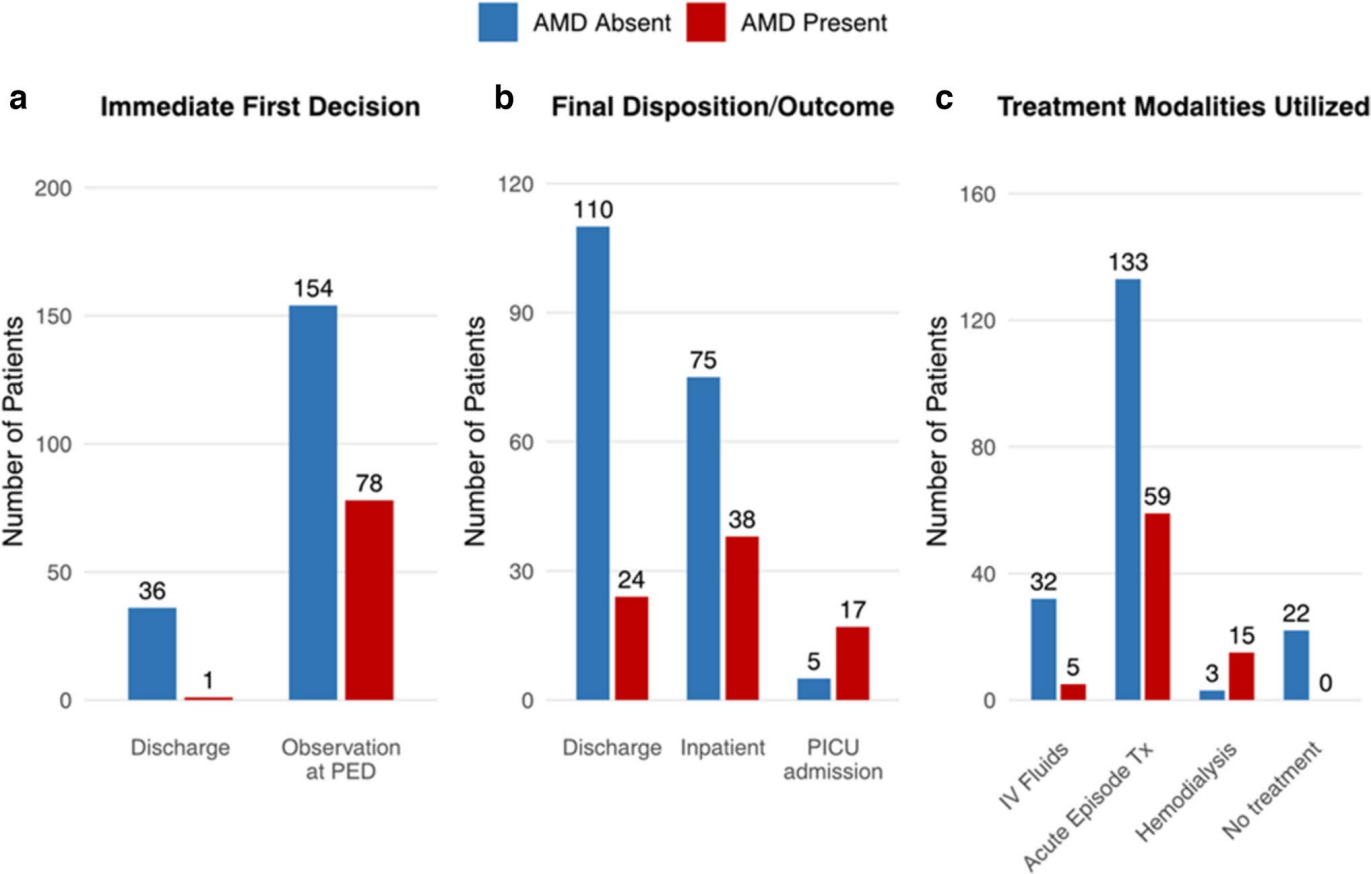
Comparison of clinical management and outcomes between pediatric emergency department visits with and without AMD in patients with MSUD. **A** Immediate first decision at presentation, **B** final disposition/outcome, **C** treatment modalities utilized

AMD significantly affected the clinical course. Compared with non-AMD visits, AMD visits required longer ED observation (median 2 vs. 1 day, *p* = 0.005), had lower discharge rates (30% vs. 58%), and higher pediatric intensive care unit admissions (22% vs. 2.6%, *p* < 0.001). Hemodialysis was required in 19% of AMD visits compared with 1.6% of non-AMD visits (*p* < 0.001), reflecting greater severity of metabolic instability (Figure 2).

### Distribution of laboratory findings according to AMD status

Several laboratory parameters differed significantly between emergency visits with and without AMD (Table 2).

**Table 2:** Laboratory findings at pediatric emergency department admission by AMD status

	*N*	AMD absent^1^	AMD present^a^	*p*-value
Ammonia, µmol/L	41	36 (9–129)	49 (26–194)	0.092^b^
Lactate, mmol/L	48	17 (1–48)	16 (7–76)	0.537^b^
pH	168	7.40 (7.19–7.60)	7.39 (7.10–7.60)	0.230^b^
pCO_2_, mmHg	169	35 (18–55)	35 (23–48)	0.666^b^
HCO_3_, mmol/L	166	22.1 (8.6–28.8)	22.2 (11.6–25.7)	0.797^b^
Glucose, mg/dL	236	102 (31–178)	92 (51–175)	** < 0.001**^**b**^
Sodium, mEq/L	241	138.0 (131.0–154.0)	136.0 (125.0–144.0)	**0.001**^**b**^
Potassium, mEq/L	241	4.50 (3.60–5.90)	4.50 (3.40–5.80)	0.817^b^
Chloride, mEq/L	241	101.0 (92.0–138.0)	100.0 (86.0–114.0)	**0.003**^**b**^
Phosphorus, mg/dL	238	4.60 (2.40–6.60)	4.60 (2.01–7.70)	> 0.999^b^
Anion gap, mEq/L	162	18.4 (4.8–36.0)	17.3 (8.5–34.0)	0.475^b^
Urea, mg/dL	242	26 (8–60)	29 (6–74)	0.110^b^
Creatinine, mg/dL	240	0.34 (0.10–0.73)	0.36 (0.18–0.89)	**0.029**^**b**^
Uric acid, mg/dL	231	3.40 (1.10–9.60)	5.10 (1.00–10.80)	** < 0.001**^**b**^
AST, U/L	240	28 (10–492)	32 (10–745)	**0.016**^**b**^
ALT, U/L	241	19 (2–1165)	22 (3–1270)	**0.044**^**b**^
Valine, µmol/L	161	498 (33–5059)	610 (122–2,696)	**0.004**^**b**^
Leucine, µmol/L	162	126 (8–587)	737 (36–3678)	** < 0.001**^**b**^
Isoleucine, µmol/L	161	293 (16–1272)	356 (33–1422)	**0.038**^**b**^
Glutamine, µmol/L	144	418 (103–962)	367 (175–976)	**0.034**^**b**^
Alanine, µmol/L	144	292 (92–902)	179 (63–642)	** < 0.001**^**b**^
Urine ketone	224			** < 0.001**^**c**^
NEGATİF		125 (80)	33 (49)	
+ 1		14 (8.9)	13 (19)	
+ 2		12 (7.6)	5 (7.5)	
+ 3		6 (3.8)	13 (19)	
+ 4		0 (0)	3 (4.5)	

^a^Median (min–max); *n* (%), ^b^Mann-Whitney *U* test, ^c^Fisher’s exact test

Bold values indicate statistically significant *p*-values (*p* < 0.05)

*AST*, aspartate aminotransferase; *ALT*, alanine aminotransferase; *AMD*, acute metabolic decompensation

Among categorical variables, hyponatremia was more frequent in AMD visits (32% vs. 16%, *p* = 0.005). Hyperuricemia showed the strongest association, occurring in 51% of AMD visits compared with 10% of non-AMD visits (*p* < 0.001). Amino acid abnormalities were also notable: hyperleucinemia was detected in 67% vs. 12% (*p* < 0.001), hypervalinemia in 78% vs. 63% (*p* = 0.047), and low alanine in 25% vs. 6.7% (*p* = 0.002). Ketonuria (51% vs. 20%, *p* < 0.001) and positive DNPH testing (64% vs. 46%, *p* = 0.044) were also more common during AMD.

Continuous measurements revealed consistent differences. Compared with non-AMD visits, AMD visits had lower glucose (median 92 vs. 102 mg/dL, *p* < 0.001), sodium (136.0 vs. 138.0 mEq/L, *p* = 0.001), and chloride levels (100.0 vs. 101.0 mEq/L, *p* = 0.003), while uric acid was markedly higher (5.10 vs. 3.40 mg/dL, *p* < 0.001). Liver enzymes were modestly elevated, with higher AST (32 vs. 28 U/L, *p* = 0.016) and ALT (22 vs. 19 U/L, *p* = 0.044).

Branched-chain amino acids showed pronounced differences: leucine (737 vs. 126 µmol/L, *p* < 0.001), valine (610 vs. 498 µmol/L, *p* = 0.004), and isoleucine (356 vs. 293 µmol/L, *p* = 0.038) were higher in AMD visits. In contrast, alanine (179 vs. 292 µmol/L, *p* < 0.001) and glutamine (367 vs. 418 µmol/L, *p* = 0.034) were lower. Higher-grade ketonuria (+3/+4) was significantly more frequent in AMD visits (*p* < 0.001).

### Clinical and laboratory predictors of AMD at emergency admission

Because elevated leucine levels are central to AMD pathophysiology and were included in the criteria for identifying AMD episodes, leucine was excluded from discriminative and predictive analyses. Correlation analyses showed moderate associations between leucine, uric acid and alanine, consistent with their shared relationship to metabolic instability. Detailed results are provided in Supplementary Material 3.

**Fig. 3:**
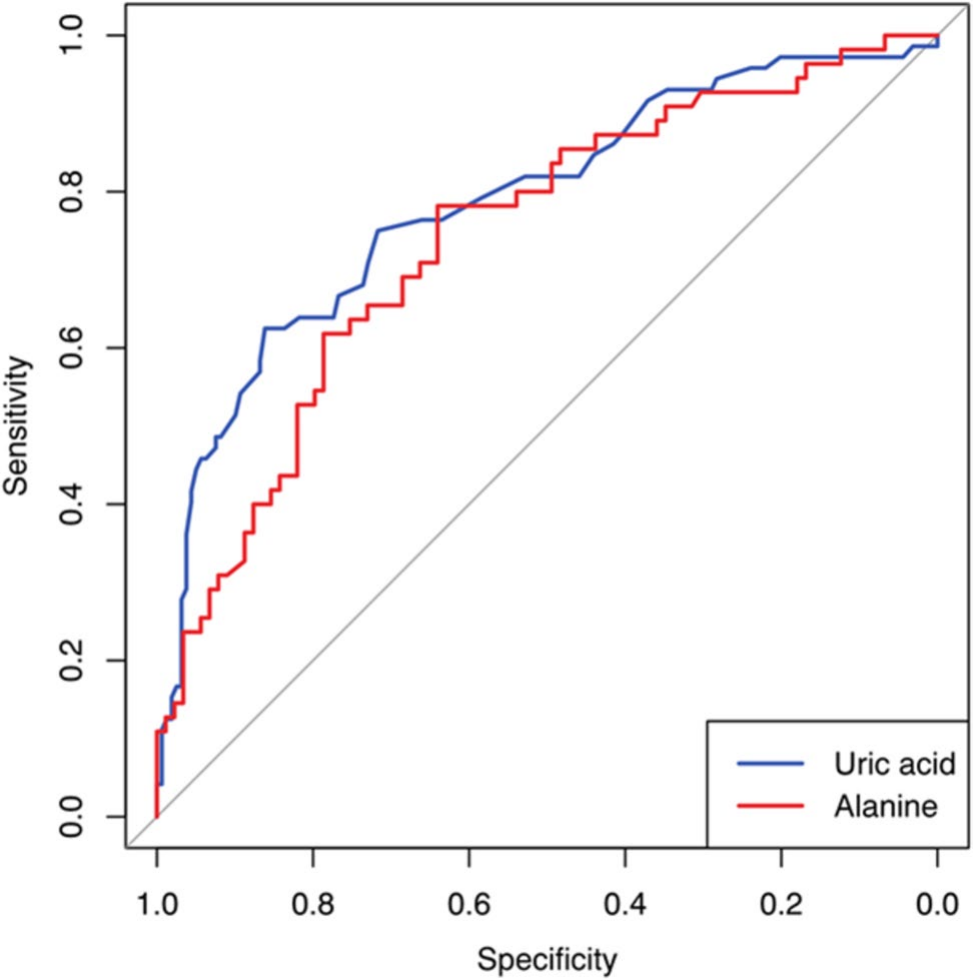
ROC curves illustrating the discriminative performance of uric acid and alanine for predicting AMD at pediatric emergency department admission

ROC curve analysis assessed the performance of other readily available laboratory parameters (Table 3, Figure 3). Serum uric acid demonstrated good discriminative ability (AUC 0.786, 95% CI 0.720–0.853, *p* < 0.001) with excellent calibration (Brier score 0.161; H-L *p* = 0.567). Plasma alanine also performed well (AUC 0.741, 95% CI 0.658–0.825, *p* < 0.001; Brier score 0.199; H-L *p* = 0.736).

**Table 3:**
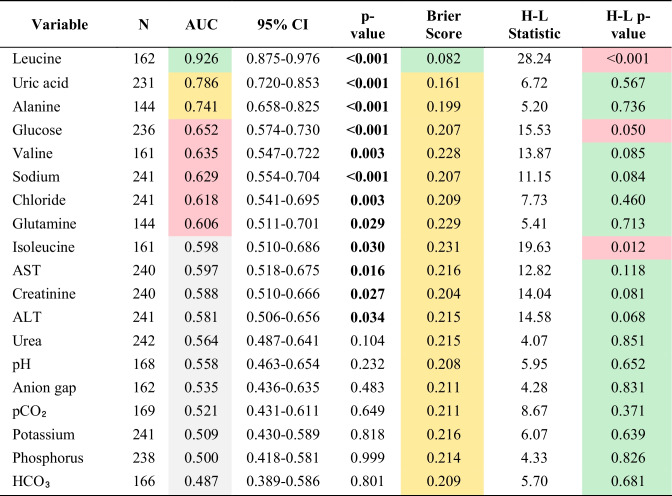
Comprehensive ROC analysis and calibration metrics for acute metabolic decompensation status

Color coding: Green, good; yellow, acceptable; red, poor. Brier score: lower is better (0, perfect; 0.25, no discrimination); H–L *p*-value > 0.05 indicates good calibration. AUC, area under the curve; CI, confidence interval; H–L: Hosmer–Lemeshow test; AST, aspartate aminotransferase; ALT, alanine aminotransferase

Moderate discrimination was observed for glucose (AUC 0.652, *p* < 0.001), valine (AUC 0.635, *p* = 0.003), sodium (AUC 0.629, *p* < 0.001), and chloride (AUC 0.618, *p* = 0.003). Glutamine, isoleucine, AST, creatinine, and ALT showed modest but significant performance (AUC 0.581–0.606). Acid–base parameters (pH, pCO_2_, HCO_3_^−^, anion gap) were not discriminative (AUC 0.487–0.558, all *p* > 0.05).

Independent predictors were assessed using Firth’s penalized logistic regression (Supplementary Material S4). To improve clinical interpretability, alanine was dichotomized at the optimal ROC-derived cutoff (249.5 µmol/L) identified in Supplementary Material S5. Of 269 visits, 126 with complete data were included; 143 (53.2%) were excluded due to missing variables. After adjustment for age and sex, neurological symptoms were the strongest predictor of AMD (HR 151.5, 95% CI 11.18–25.75, *p* < 0.001), while respiratory symptoms were inversely associated (HR 0.189, 95% CI 0.03–0.80, *p* = 0.022).

Among laboratory parameters, serum uric acid remained independently predictive (HR 2.012, 95% CI 1.213–3.760, *p* = 0.005), indicating that each 1 mg/dL increase nearly doubled the likelihood of AMD. Plasma alanine also remained significant (HR 4.062, 95% CI 1.218–15.59, *p* = 0.022), with lower levels associated with higher AMD risk. Despite strong univariate performance, valine was not significant in the multivariable model, suggesting collinearity with other metabolic parameters. Age, sex, fever, glucose, and valine were not independent predictors in the final model.

Dehydration and reduced oral intake at emergency presentation may affect creatinine levels and could theoretically contribute to elevated uric acid concentrations. Therefore, additional analyses were conducted to examine the potential effect of renal function on uric acid levels. The overall correlation between uric acid and creatinine was weak (ρ = 0.32, *p* < 0.001) and remained similarly weak when stratified by AMD status (non-AMD: *ρ* = 0.30, *p* < 0.001; AMD: *ρ* = 0.29, *p* = 0.016). The consistency of these low correlation coefficients (<0.4) across groups suggests that uric acid elevation was not primarily related to reduced renal clearance. BUN-to-creatinine ratios were similar between groups (median 37.1 in non-AMD vs. 34.0 in AMD visits, *p* = 0.214). Stratified ROC analyses showed that uric acid maintained strong discriminative performance across creatinine quartiles. The AUC was 0.804 in the lowest creatinine quartile and 0.856 in the highest quartile, with no significant difference between strata (DeLong test *p* = 0.468). In multivariable Firth logistic regression models adjusted for creatinine, uric acid remained an independent predictor of AMD (OR 2.19, 95% CI 1.27–4.36, *p* = 0.003). The effect size was similar to the model without creatinine adjustment (OR 2.01, *p* = 0.007). Creatinine itself was not significantly associated with AMD (OR 0.03, 95% CI 0.00–32.38, *p* = 0.335). ROC analyses stratified by creatinine quartiles and multivariable Firth logistic regression models adjusted for creatinine are presented in Supplementary Material S6.

### Cutoff values of biomarkers predicting AMD

Optimal cutoff values for the two best-performing biomarkers were determined using Youden’s index (Supplementary Material S5).

For serum uric acid, a threshold of 4.65 mg/dL yielded 62% sensitivity and 86% specificity, with a positive predictive value (PPV) of 0.67, negative predictive value (NPV) of 0.84, and overall accuracy of 79%. The positive (LR +) and negative (LR −) likelihood ratios were 4.5 and 0.4, respectively, indicating good rule-in performance.

For plasma alanine, a cutoff of 249.50 µmol/L provided 78% sensitivity and 64% specificity, with a PPV of 0.57, NPV of 0.83, and overall accuracy of 69%. The corresponding likelihood ratios (LR + 2.2; LR − 0.3) suggest greater utility as a rule-out parameter.

## Discussion

The primary objective of this study was to identify rapid, readily accessible predictors to facilitate early recognition of AMD in children with MSUD presenting to the pediatric ED. By analyzing 269 emergency visits from 25 MSUD patients, 29.4% of which were associated with AMD, we provide one of the most comprehensive evaluations of AMD presentation, laboratory profiles, and short-term outcomes in acute care settings. Importantly, no single clinical symptom reliably distinguished AMD from non-AMD presentations at admission. While nonspecific complaints such as vomiting, decreased oral intake, and malaise were common in both groups, respiratory symptoms and fever were more frequent in non-AMD visits, suggesting that infectious features alone do not necessarily indicate metabolic instability. AMD was associated with worse clinical outcomes, including longer emergency observation, lower discharge rates, and a significantly higher need for intensive care and hemodialysis. A major strength of this study is its detailed assessment of primary laboratory biomarkers available at initial evaluation. Among multiple biochemical abnormalities observed during AMD, serum uric acid and plasma alanine demonstrated the strongest discriminative performance. Notably, hyperuricemia and low alanine levels remained significant after adjustment for age, sex, and other laboratory variables, and clinically applicable cutoff values were identified (4.65 mg/dL for uric acid and 249.50 µmol/L for alanine). In emergency settings where rapid plasma leucine testing is not feasible, hyperuricemia may serve as a practical surrogate for early AMD risk assessment.

AMD is the most critical complication of MSUD and typically occurs during catabolic stress despite ongoing metabolic monitoring. It is characterized by rapid accumulation of BCAAs, particularly leucine and their α-ketoacids, leading to acute metabolic intoxication and neurological dysfunction. Elevated plasma leucine plays a central neurotoxic role and is directly associated with encephalopathy, cerebral edema, and death if untreated [18]. Neurotoxicity results from the accumulation of leucine and its metabolites due to BCKAD dysfunction [4], leading to osmotic cellular swelling, impaired large neutral amino acid transport across the blood–brain barrier, mitochondrial dysfunction, oxidative stress, and altered neurotransmitter synthesis [19]. These processes may be exacerbated by disturbances in sodium and osmolality, further worsening cerebral edema [12].

The clinical consequences of AMD are profound. In our cohort, AMD was associated with increased healthcare utilization and need for extracorporeal detoxification, reflecting the substantial burden of AMD on both patient prognosis and healthcare resources. Beyond acute morbidity, recurrent or poorly controlled episodes negatively affect long-term neurodevelopment. Longitudinal studies have consistently shown that early treatment of hyperleucinemic episodes reduces neurological injury [20], and sustained elevations in leucine and frequent AMD episodes correlate with lower cognitive outcomes [21]. These findings underscore the importance of early recognition and timely intervention.

Taken together, these observations highlight the critical importance of early recognition and effective management of AMD, ideally before severe neurological manifestations develop. Although therapeutic strategies for AMD are well established, early diagnosis remains challenging. Neurological manifestations often occur late, after significant metabolic injury has already occurred. Initial symptoms are typically nonspecific and overlap with common childhood infections, complicating emergency decision-making. Furthermore, plasma leucine measurement—the primary diagnostic biomarker—is not universally or rapidly available, creating uncertainty about when to initiate intensive metabolic therapy.

Plasma leucine remains the principal toxic metabolite in MSUD. Concentrations above 600 μmol/L are associated with cognitive and motor deterioration, and levels exceeding 1000 μmol/L may precipitate encephalopathy and coma [19]. Both absolute levels and fluctuations in leucine contribute to neurological risk, reflecting the narrow therapeutic margin in MSUD management. During AMD, leucine elevation is accompanied by broader catabolic disturbances, including increased ketones, anion gap, lactate, ammonia, and uric acid, along with decreased glucose, sodium, and chloride [19, 22]. However, data identifying which of these parameters independently predict AMD at presentation remain limited. The only prior emergency-based predictor study by Yıldız et al. evaluated 115 visits from 29 MSUD patients and identified poor feeding, fatigue, elevated uric acid, and increased anion gap as associated with AMD [15]. Beyond this single report, systematic evaluation of rapidly obtainable laboratory predictors has been scarce. In our study, although several laboratory parameters differed between groups in univariate analyses, serum uric acid was the only independent predictor after multivariable adjustment. This suggests that while AMD produces widespread biochemical changes, not all abnormalities have equivalent predictive value at emergency presentation. Importantly, we identified a clinically applicable uric acid cutoff (4.65 mg/dL) with favorable diagnostic performance, providing actionable guidance for early risk stratification. Additional analyses demonstrated that the association between uric acid and AMD was not driven by renal dysfunction. Uric acid retained its predictive value after adjustment for creatinine and showed consistent discriminative performance across different creatinine levels. These findings support the interpretation that hyperuricemia primarily reflects increased catabolic stress rather than impaired renal clearance. Plasma alanine also demonstrated meaningful discriminative value and likely reflects increased protein turnover and catabolic stress during decompensation.

Hyperleucinemia is the fundamental biochemical basis of AMD in MSUD; however, a single leucine measurement taken at emergency presentation may underestimate an evolving metabolic crisis. In our cohort, all biochemical samples were collected immediately upon ED admission, before initiation of treatment. All patients had previously received structured sick-day education encouraging very early hospital presentation, often at the onset of decreased intake, ketonuria, or mild neurological symptoms. Early presentation may therefore capture the initial phase of catabolic deterioration, before leucine reaches peak concentrations. Metabolic kinetic studies have shown that plasma leucine increases during illness may develop progressively over hours to days, rather than instantaneously, with reduced intake and fasting contributing substantially to leucine accumulation [23]. Experimental models further suggest that neurological injury in MSUD may also involve branched-chain ketoacid–mediated disruption of cerebral energy metabolism in addition to leucine neurotoxicity [24]. Within this framework, serum uric acid may provide additional clinical value as an early indicator of catabolic stress, particularly when plasma leucine has not yet reached markedly elevated levels at the time of first assessment.

This study has limitations. Its retrospective, single-center design may limit generalizability. Chronic valine and isoleucine supplementation may have influenced measured amino acid levels, potentially reducing their predictive value. Additionally, the absence of serial leucine measurements prevented assessment of dynamic changes during emergency presentations. Prospective, multicenter studies with standardized sampling and longitudinal amino acid monitoring are needed to validate these findings.

## Conclusion

Our findings expand the limited literature by showing that, among routinely available laboratory parameters, serum uric acid is the most reliable independent predictor of AMD in pediatric MSUD patients, while plasma alanine provides complementary insight into the underlying catabolic state. This combined laboratory approach offers a practical strategy for early risk stratification, helping bridge the gap between nonspecific clinical presentation and delayed definitive biochemical confirmation.

## Supplementary Information

Below is the link to the electronic supplementary material.

Supplementary file1 Age distribution and feeding modalities of MSUD patients presenting to the pediatric emergency department (DOCX 12.9 KB)

Supplementary file2 Demographic and genotypic characteristics of the MSUD patients included in the study (DOCX 16.6 KB)

Supplementary file3 Correlation analysis between uric acid and amino acids (DOCX 15.8 KB)

Supplementary file4 Multivariate analysis of risk factors for AMD status (DOCX 13.9 KB)

Supplementary file5 Diagnostic performance of serum biomarkers for discriminating acute metabolic decompensation status (DOCX 13.9 KB)

Supplementary file6 ROC analyses stratified by creatinine quartiles and multivariable Firth logistic regression models adjusted for creatinine (DOCX 174 KB)

## Data Availability

All data relevant to the study are included in the article. The corresponding author is the guarantor for any additional information or any queries regarding the article and could be reached through any of the contact information given.

## Abbreviations

AVPU: Alert, verbal, pain, unresponsive
MSUD: Maple syrup urine disease
BCKAD: Branched-chain α-keto acid dehydrogenase
BCAA: Branched-chain amino acid
BCKDHA: Subunits E1α
BCKDHB: Subunits E1β
DBT: Subunits E2
DLD: Subunits E3
AMD: Acute metabolic decompensation
ED: Emergency department
ALT: Alanine aminotransferase
AST: Aspartate aminotransferase
ALP: Alkaline phosphatase
GGT: Gamma-glutamyl transferase
DNPH: Dinitrophenylhydrazine
ROC: Receiver operating characteristic
AUC: Area under the curve
VIF: Variance inflation factor
PPV: Positive predictive value
NPV: Negative predictive value
LR +: Positive likelihood
LP −: Negative likelihood
