# Supplementary material for: Early laboratory indicators of acute metabolic decompensation during emergency presentations in pediatric maple syrup urine disease
Source: Eur J Pediatr. 2026 May 19;185(6):412. doi: 10.1007/s00431-026-07081-4 (PMC13183725; doi:10.1007/s00431-026-07081-4)
Supplement: Supplementary file 1 — Supplementary file1 Age distribution and feeding modalities of MSUD patients presenting to the pediatric emergency department (DOCX 12.9 KB) [file 431_2026_7081_MOESM1_ESM.docx]

**Supplementary Material S1: Age distribution and feeding modalities of MSUD patients presenting to the pediatric emergency department**

|  | **All PED visits**  N = 269^1^ | **AMD absent**   N = 190^1^ | **AMD present**   N = 79^1^ | **p-value** |
| --- | --- | --- | --- | --- |
| **Age** | 4.3 (0.0-20.0) | 4.1 (0.0-20.0) | 4.9 (0.0-19.6) | 0.122^2^ |
| **Age groups** |  |  |  | 0.253^3^ |
| <5 years | 154 (57) | 113 (59) | 41 (52) |  |
| ≥5 years | 115 (43) | 77 (41) | 38 (48) |  |
| **Feeding route** |  |  |  | 0.105^4^ |
| PO | 238 (88) | 163 (86) | 75 (95) |  |
| PEG | 29 (11) | 25 (13) | 4 (5.1) |  |
| NG | 2 (0.7) | 2 (1.1) | 0 (0) |  |
| \| ^1^Median (Min-max); n (%), ^2^Mann-Whitney U test, ^3^Pearson’s Chi-squared test, ^4^Fisher’s exact test  *PED, pediatric emergency department; AMD, acute metabolic decompensation; PO, per oral; PEG, percutaneous endoscopic gastrostomy; NG, nasogastric* \| \| --- \| | | | | |
