# Supplementary material for: Early laboratory indicators of acute metabolic decompensation during emergency presentations in pediatric maple syrup urine disease
Source: Eur J Pediatr. 2026 May 19;185(6):412. doi: 10.1007/s00431-026-07081-4 (PMC13183725; doi:10.1007/s00431-026-07081-4)
Supplement: Supplementary file 2 — Supplementary file2 Demographic and genotypic characteristics of the MSUD patients included in the study (DOCX 16.6 KB) [file 431_2026_7081_MOESM2_ESM.docx]

| **Supplementary Material S2: Demographic and genotypic characteristics of the MSUD patients included in the study.** | | | | | | |
| --- | --- | --- | --- | --- | --- | --- |
| **Patient ID** | **Sex** | **Gene** | **Nucleotide change** | **Protein change** | **Age of diagnosis (days)** | **Current age (years)** |
| 1 | Female | *BCKDHB* | c.93_103del | p. (Ala32Phefs*48) | 7 | 21 |
| 2 | Male | *BCKDHA* | c. 452C>T | p.(Thr151Met) | 343 | 18 |
| 3 | Male | BCKDHB | c.564T>A | p.(Cys188*) | 20 | 21 |
| 4 | Female | *np^*^* |  |  | 5 | 25 |
| 5 | Male | BCKDHA | c.773_774del | p.(Cys258Serfs*19) | 21 | 17 |
| 6 | Male | *np^*^* |  |  | 180 | 14 |
| 7 | Female | *np^*^* |  |  | 720 | 8 |
| 8 | Female | BCKDHB | c.93_103del | p. (Ala32Phefs*48) | 3 | 14 |
| 9 | Male | BCKDHB | c.1149T>A | p.(Tyr383*) | 135 | 12 |
| 10 | Male | *np^*^* |  |  | 5 | 13 |
| 11 | Female | BCKDHA | c.641delC | p.( Pro214Leufs*116) | 27 | 3 |
| 12 | Female | *BCKDHA* | c.773_774delinsAA | p.(Cys258*) | 15 | 10 |
| 13 | Male | *np^*^* |  |  | 5 | 10 |
| 14 | Male | *BCKDHA* | c.773_774delinsAA | p.(Cys258*) | 7 | 10 |
| 15 | Female | *BCKDHB* | c.169C>T | p.(Gln57*) | 12 | 10 |
| 16 | Male | *BCKDHB* | c.1149T>A | p.(Tyr383*) | 9 | 9 |
| 17 | Male | *BCKDHB* | c.633+1G>T |  | 17 | 9 |
| 18 | Male | BCKDHB | c.508C>G | p.(Arg170Gly) | 15 | 9 |
| 19 | Male | *BCKDHA* | c.773_774delinsAA | p.(Cys258*) | 23 | 8 |
| 20 | Male | BCKDHA | c.641delC | p.( Pro214Leufs*116) | 1 | 8 |
| 21 | Female | *np^*^* |  |  | 1295 | 7 |
| 22 | Male | *np^*^* |  |  | 32 | 9 |
| 23 | Female | BCKDHB | c.331C>T | p.(Arg111*) | 20 | 7 |
| 24 | Male | *BCKDHB* | c.1149T>A | p.(Tyr383*) | 6 | 7 |
| 25 | Male | *np^*^* |  |  | 38 | 3 |
| ^*^*Diagnosis was made based on biochemical evidence of increased plasma levels of leucine, isoleucine, valine and alloisoleucine, as well as corresponding α-ketoacids in urine.* | | | | | | |
