# Supplementary material for: Early laboratory indicators of acute metabolic decompensation during emergency presentations in pediatric maple syrup urine disease
Source: Eur J Pediatr. 2026 May 19;185(6):412. doi: 10.1007/s00431-026-07081-4 (PMC13183725; doi:10.1007/s00431-026-07081-4)
Supplement: Supplementary file 3 — Supplementary file3 Correlation analysis between uric acid and amino acids (DOCX 15.8 KB) [file 431_2026_7081_MOESM3_ESM.docx]

**Supplementary Material S3: Correlation analysis between uric acid and amino acids**

| **Laboratory Parameter** | **Uric acid** | **Leucine** | **Valine** | **Isoleucine** | **Glutamine** | **Alanine** |
| --- | --- | --- | --- | --- | --- | --- |
| Uric acid | 1.00 | 0.57^***^ | 0.13 | 0.07 | -0.14 | -0.37^***^ |
| Leucine | 0.57^***^ | 1.00 | 0.13 | 0.16^*^ | -0.15 | -0.40^***^ |
| Valine | 0.13 | 0.13 | 1.00 | 0.56^***^ | -0.08 | -0.17^*^ |
| Isoleucine | 0.07 | 0.16^*^ | 0.56^***^ | 1.00 | -0.02 | -0.09 |
| Glutamine | -0.14 | -0.15 | -0.08 | -0.02 | 1.00 | 0.47^***^ |
| Alanine | -0.37^***^ | -0.40^***^ | -0.17* | -0.09 | 0.47^***^ | 1.00 |
| *p < 0.05; ^**^ p < 0.01; ^***^ p < 0.001.* | | | | | | |
| *Correlation strength (absolute value of ρ): 0.00–0.19 (Very Weak); 0.20–0.39 (Weak); 0.40–0.59 (Moderate); 0.60–0.79 (Strong); 0.80–1.00 (Very Strong).* | | | | | | |
