# Supplementary material for: Early laboratory indicators of acute metabolic decompensation during emergency presentations in pediatric maple syrup urine disease
Source: Eur J Pediatr. 2026 May 19;185(6):412. doi: 10.1007/s00431-026-07081-4 (PMC13183725; doi:10.1007/s00431-026-07081-4)
Supplement: Supplementary file 4 — Supplementary file4 Multivariate analysis of risk factors for AMD status (DOCX 13.9 KB) [file 431_2026_7081_MOESM4_ESM.docx]

**Supplementary Material S4: Multivariate analysis of risk factors for AMD status**

| **Variable*** | **OR (95% CI)** | **p-value** |
| --- | --- | --- |
| Age | 1.031 (0.911-1.171) | 0.624 |
| Female sex | 1.130 (0.267-4.648) | 0.864 |
| Fever | 0.360 (0.054-1.802) | 0.221 |
| Respiratory tract symptoms | 0.189 (0.030-0.800) | **0.022** |
| Neurological symptoms | 151.5 (11.18-25,750) | **<0.001** |
| Glucose | 0.996 (0.955-1.033) | 0.824 |
| Uric acid | 2.012 (1.213-3.760) | **0.005** |
| Valine | 1.000 (0.998-1.001) | 0.808 |
| Alanine<249.5 µmol/L | 4.062 (1.218-15.59) | **0.022** |
| OR: Odds ratio, CI: Confidence interval  **Analysis includes 126 observations with complete covariate data; 143 observations excluded due to missing values (total N=269)* | | |
