# Supplementary material for: Early laboratory indicators of acute metabolic decompensation during emergency presentations in pediatric maple syrup urine disease
Source: Eur J Pediatr. 2026 May 19;185(6):412. doi: 10.1007/s00431-026-07081-4 (PMC13183725; doi:10.1007/s00431-026-07081-4)
Supplement: Supplementary file 5 — Supplementary file5 Diagnostic performance of serum biomarkers for discriminating acute metabolic decompensation status (DOCX 13.9 KB) [file 431_2026_7081_MOESM5_ESM.docx]

**Supplementary Material S5: Diagnostic performance of serum biomarkers for discriminating acute metabolic decompensation status**

| **Parameters** | **Optimum**  **cut-off** | **Sensitivity** | **Specificity** | **LR+** | **LR-** | **PPV** | **NPV** | **Accuracy** |
| --- | --- | --- | --- | --- | --- | --- | --- | --- |
| Uric acid | 4.65 | 0.62 | 0.86 | 4.5 | 0.4 | 0.67 | 0.84 | 0.79 |
| Alanine | 249.50 | 0.78 | 0.64 | 2.2 | 0.3 | 0.57 | 0.83 | 0.69 |
| Leucine^*^ | 606.60 | 0.80 | 1.00 | Inf | 0.2 | 1.00 | 0.89 | 0.93 |
| *Leucine was not included in multivariable models because it was part of the AMD definition and showed collinearity with other biochemical markers.  *LR+: Positive likelihood ratio, LR-: Negative likelihood ratio, PPV: Positive predictive value, NPV: Negative predictive value, AUC: Area under the curve* | | | | | | | | |
