# Supplementary material for: Early laboratory indicators of acute metabolic decompensation during emergency presentations in pediatric maple syrup urine disease
Source: Eur J Pediatr. 2026 May 19;185(6):412. doi: 10.1007/s00431-026-07081-4 (PMC13183725; doi:10.1007/s00431-026-07081-4)
Supplement: Supplementary file 6 — Supplementary file6 ROC analyses stratified by creatinine quartiles and multivariable Firth logistic regression models adjusted for creatinine (DOCX 174 KB) [file 431_2026_7081_MOESM6_ESM.docx]

**Supplementary Material S6: Multivariate analysis of risk factors for AMD status adjusted for creatinine**

| **Variable*** | **OR (95% CI)** | **p-value** |
| --- | --- | --- |
| Age | 1.093 (0.923-1.317) | 0.307 |
| Female sex | 1.248 (0.285-5.393) | 0.764 |
| Fever | 0.416 (0.060-2.203) | 0.310 |
| Respiratory tract symptoms | 0.201 (0.031-0.870) | **0.031** |
| Neurological symptoms | 170.5 (12.11-30,240) | **<0.001** |
| Glucose | 0.992 (0.951-1.031) | 0.708 |
| Creatinine | 0.032 (0.000-32.38) | 0.335 |
| Uric acid | 2.189 (1.266-4.362) | **0.003** |
| Valine | 1.000 (0.998-1.002) | 0.912 |
| Alanine<249.5 µmol/L | 3.800 (1.133-14.64) | **0.030** |
| OR: Odds ratio, CI: Confidence interval  **Analysis includes 126 observations with complete covariate data; 143 observations excluded due to missing values (total N=269)* | | |


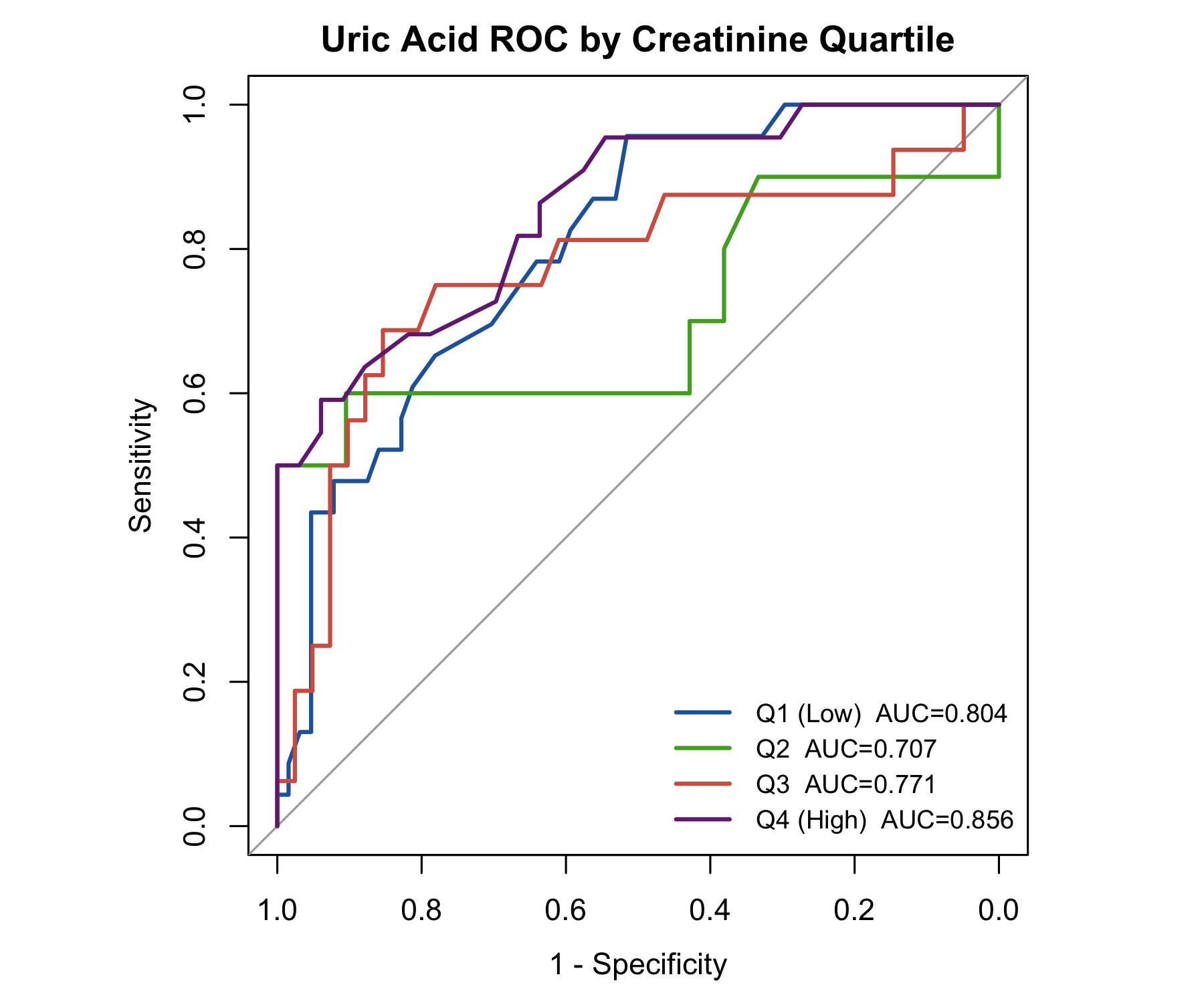


**Figure: Uric acid ROC curves comparison between creatinine quartiles. Q1 is not significantly different from Q4 (p = 0.468)**
